# Smoking-promoted oxidative DNA damage response is highly correlated to lung carcinogenesis

**DOI:** 10.18632/oncotarget.7810

**Published:** 2016-03-01

**Authors:** Chao Cao, Tianwen Lai, Miao Li, Hongbin Zhou, Dan Lv, Zaichun Deng, Songmin Ying, Zhihua Chen, Wen Li, Huahao Shen

**Affiliations:** ^1^ Department of Respiratory and Critical Care Medicine, Second Affiliated Hospital, Zhejiang University School of Medicine, Hangzhou, China; ^2^ Department of Respiratory Medicine, Affiliated Hospital of School of Medicine of Ningbo University, Ningbo, China; ^3^ State Key Laboratory for Respiratory Diseases, Guangzhou, China

**Keywords:** lung cancer, smoking, oxidative DNA damage, 8-OHdG, biomarker

## Abstract

Oxidative stress induced by tobacco smoking is one of the main causes of DNA damage and is known to be involved in various cancers. Smoking is the leading cause of lung cancer, while the role of cigarette smoke-induced oxidative DNA damage response during lung carcinogenesis is largely unknown. In this study, we investigated oxidative DNA damage response levels in smoking and nonsmoking patients with lung cancer, and evaluated the potential diagnostic value of 8-OHdG for lung cancer. We observed a higher level of 8-OHdG expression and secretion in airways of lung cancer patients than that of noncancer controls. 8-OHdG expression was associated with the TNM stages. Additionally, cigarette smoke-induced oxidative DNA damage response was observed in bronchial epithelial cells *in vitro* and *in vivo*. A statistical significance correlation was found between the levels of 8-OHdG and smoking index. With a cut-off value of 2.86 ng/ml, 8-OHdG showed a sensitivity and specificity of 70.0% and 73.7%, respectively, to identify a patient with lung cancer. These findings not only underscore the importance of smoking in oxidative DNA damage response of lung cancer patients, but also suggest 8-OHdG as a potential diagnostic biomarker for lung cancer.

## INTRODUCTION

Lung cancer is one of the most common form of malignant diseases and the leading cause of cancer-related mortality worldwide [1]. It is reported that approximately two-thirds of lung cancer patients is the presence of advance disease at the time of diagnosis [2]. Hence novel lung cancer diagnostic tests, which can be used to screen individuals at high risk, are required. Published evidence demonstrated that tumor-associated cytokines in lung cancer reflect immunologic reactions of the lung in pulmonary malignancies [3]. In the past decades, numerous studies have been conducted to investigate the usefulness of serum biomarkers for differential diagnosis of lung cancer. Moreover, in our recent study, we observed that levels of cancer-specific markers in bronchoalveolar lavage fluid (BALF) present at a higher concentration and elevated much earlier than those in peripheral blood [4]. Therefore, biomarkers testing in BALF may be more useful than that in serum for lung cancer [5–10].

Oxidative DNA damage is deeply involved in a great range of biological activities and disease states, such as cancer, aging, and neurodegeneration [11]. The base-excision repair (BER) pathway is the primary mechanism for repair of oxidative base lesions [11, 12]. If failed to repaired, oxidative DNA damage results in mutagenesis and genomic instability. Overall, these events may contribute to the initiation, maintenance, and progression of cancer [13]. Therefore, defects in DNA damage recognition and repair are central to the pathogenesis of human malignancies. The clearly revealing the role of oxidative DNA damage response in cancer is quite important to provide biomarkers for cancer risk assessment, early detection, and prognosis [14].

8-Hydroxydeoxyguanosine (8-OHdG) is one of the most commonly formed DNA lesions produced in response to oxidative stress and is considered as reliable biomarker for oxidative DNA damage [15, 16]. Numerous studies show that cigarette smoke causes a gradual decrease in antioxidant capacity and interfere DNA repair capacity that eventually induces oxidative DNA damage [17–20]. Tobacco smoking is the leading cause of lung cancer, while the role of cigarette smoke-induced oxidative DNA damage response in lung cancer is largely unknown. In order to explore oxidative DNA damage response in smoking and nonsmoking patients with lung cancer, we performed the present study to investigate 8-OHdG expression in airways by comparing levels of them in noncancer controls.

## RESULTS

### Cigarette smoke-induced oxidative DNA damage response in bronchial epithelial cells *in vitro* and *in vivo*

**Figure 1:**
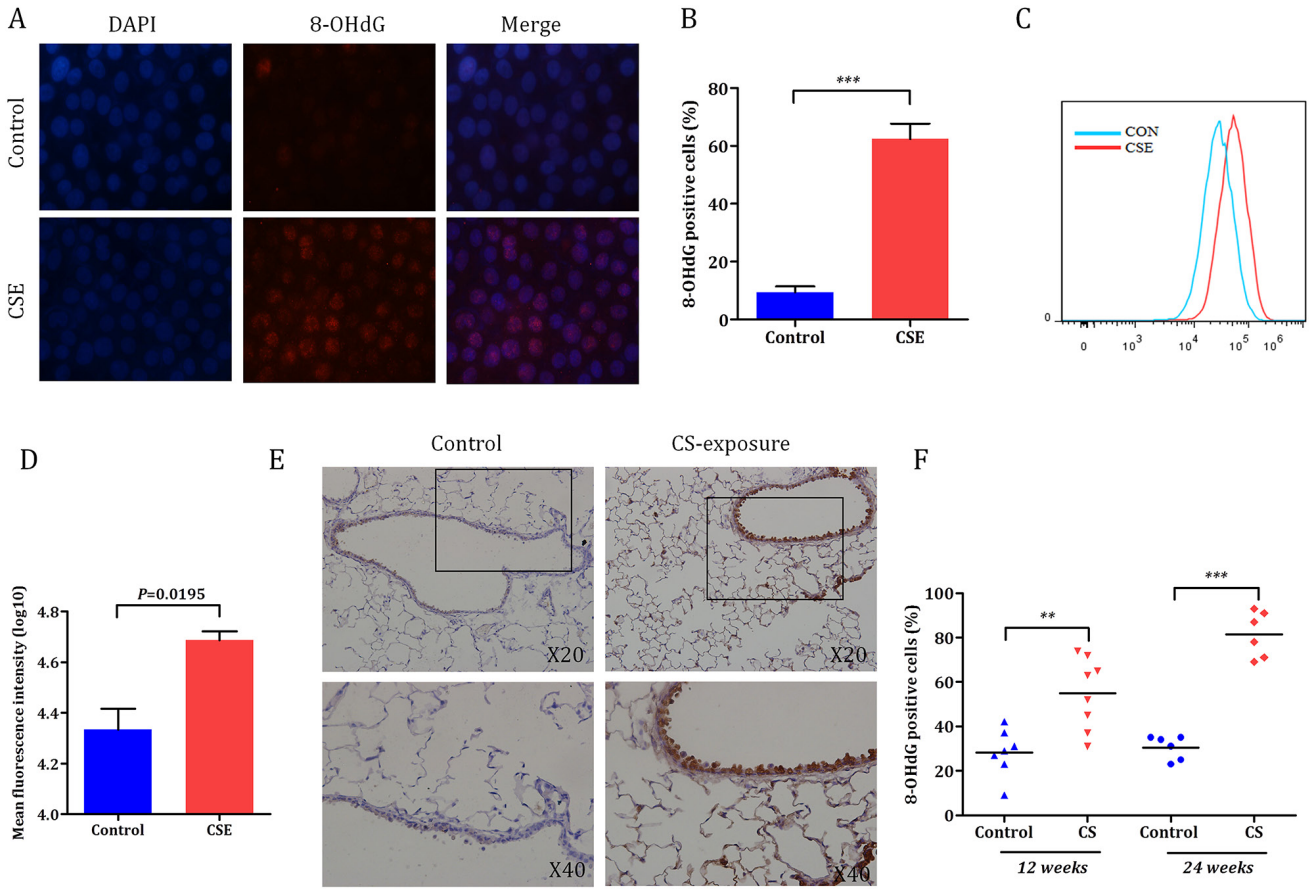
Cigarette smoke-induced oxidative DNA damage response in bronchial epithelial cells **A.** Representative microphotograph of HBE cells stained with DAPI (blue fluorescence) and anti-8-OHdG antibody (red fluorescence). **B.** Percentages of 8-OHdG positive cells in control and CSE group were counted (***P < 0.001). **C.** Oxidative DNA damage response of HBE cells treated with CSE was assessed (FACS, 8-OHdG). **D.** Significant increased positive of 8-OHdG cells was observed in CSE group than that of controls (*P* = 0.0195). **E.** C57BL/6 mice were exposed to smoke for 12 weeks and 24 weeks and oxidative DNA damage response of lung tissues was assessed (IHC, 8-OHdG). **F.** Percentages of oxidative DNA damage in control and cigarette smoke-exposure group were counted in the lung. Values in the bar graphs are given as mean ± SEM, n=6-8 mice per group; CSE, cigarette smoke extract.

To demonstrate cigarette smoke-induced oxidative DNA damage response in bronchial epithelial cells, we first detected the expression of 8-OHdG in HBE cells after treated with cigarette smoke extract (CSE) (Figure 1A). As shown in Figure 1B, significant increased positive of 8-OHdG cells was observed in CSE treatment group than that of controls (62.4 ± 5.3% *versus* 9.5 ± 2.0%, *P* = 0.0007). These results were further confirmed by flow cytometry assay (Figure 1C, 1D). *In vivo*, mice were exposed to smoke for 12 or 24 weeks and oxidative DNA damage response of the lung was assessed by immunohistochemistry (IHC) staining (Figure 1E). Significant difference in positive of 8-OHdG cells was observed between cigarette smoke-exposure group and control group (54.9 ± 5.7% *versus* 28.3 ± 4.0%, *P* = 0.0026 for 12 weeks; 81.5 ± 4.2% *versus* 30.5 ± 2.2%, *P* < 0.0001 for 24 weeks) (Figure 1F).

### Oxidative DNA damage response in lung cancer patients and noncancer controls

**Figure 2:**
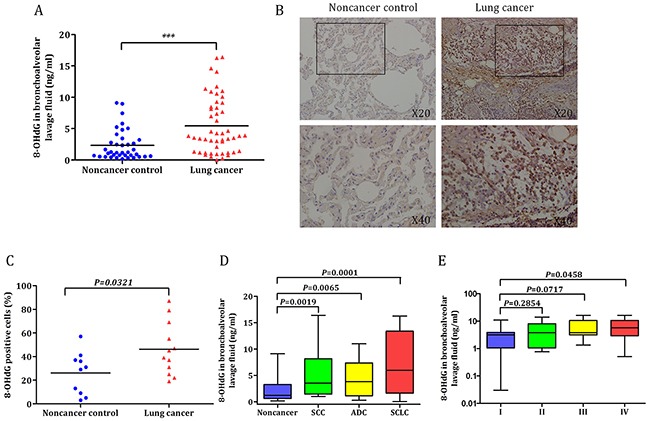
Oxidative DNA damage response in lung cancer patients and noncancer controls **A.** Comparison of 8-OHdG levels in BALF between lung cancer and noncancer group. The levels of 8-OHdG were significantly higher in lung cancer patients than those in controls (***P < 0.001). **B.** Representative examples of immunohistochemistry of 8-OHdG expression in human lung specimens. **C.** Quantification of 8-OHdG expression in the lung of smokers and nonsmokers (*P* = 0.0321). **D**. In subgroup analysis by tumor histology, 8-OHdG expression remained significantly higher in different cell types of lung cancer patients than noncancer controls. **E.** BALF 8-OHdG concentration were assessed according to TNM stage, and the levels of 8-OHdG was significantly higher in TNM IV stage patients than stage I patients. Horizontal lines in scatter plot represent the mean values. Values in the box plot are given as median (interquartile range); BALF, bronchoalveolar lavage fluid; SCC, squamous cell carcinoma; ADC, adenocarcinoma; SCLC, small-cell lung cancer.

The levels of 8-OHdG was detected in BALF of 50 lung cancer patients and 38 noncancer controls. The clinicopathological characteristics of patients were given in Table 1. As shown in Figure 2A, a significant higher 8-OHdG levels was observed among patients with lung cancer than controls (5.4 ± 0.6 ng/ml *versus* 2.3 ± 0.4 ng/ml, *P* = 0.0002). The expression of 8-OHdG in lung cancer patients and noncancer controls was further confirmed by IHC staining (Figure 2B). The percentage of 8-OHdG positive cells in lung cancer patients was significant higher than that of noncancer group (46.3 ± 6.4% *versus* 26.1 ± 5.6%, *P* = 0.0321; Figure 2C). The levels of 8-OHdG in BALF from the same 50 lung cancer patients were analyzed according to tumor histology. The pathologic types included 19 squamous cell carcinomas (SCC), 19 adenocarcinomas (ADC), and 12 small-cell lung cancer (SCLC). A statistically significant difference was found between patients with different cell type of lung cancer and noncancer individuals (5.2 ± 1.0 ng/ml for SCC, *P* = 0.0019; 4.6 ± 0.8 ng/ml for ADC, *P* = 0.0065; 7.3 ± 1.7 pg/ml for SCLC, *P* = 0.0001; Figure 2D). In addition, BALF 8-OHdG concentration was increased as the TNM stage increased, and the levels of 8-OHdG were significantly higher in TNM IV stage patients than stage I patients (6.7 ± 1.3 ng/ml *versus* 3.3 ± 0.9 ng/ml, *P* = 0.0458; Figure 2E). These findings suggested that 8-OHdG could possibly behave as a biomarker for lung cancer.

**Table 1:** Clinical characteristics of recruited participants

	Lung cancer (n=50)	Noncancer control (n=38)	*P* value
Age, years			0.135
Mean ± SD	63.0 ± 9.6	60.1 ± 7.8	
Range	37-81	45-79	
Gender, n (%)			0.765
Male	42 (84%)	31 (81.6%)	
Female	8 (16%)	7 (18.4%)	
Smoking status, n (%)			0.888
Smokers	31 (62%)	23 (60.5%)	
Nonsmokers	19 (38%)	15 (39.5%)	
Pack-years	54.8 ± 6.3	41.4 ± 5.6	0.134
Cell type, n (%)			
Squamous cell carcinoma	19 (38%)		
Adenocarcinoma	19 (38%)		
Small-cell lung cancer	12 (24%)		
TNM stage, n (%)			
I	12 (24%)		
II	12 (24%)		
III	12 (24%)		
IV	14 (28%)		

### Oxidative DNA damage response in smokers and nonsmokers

**Figure 3:**
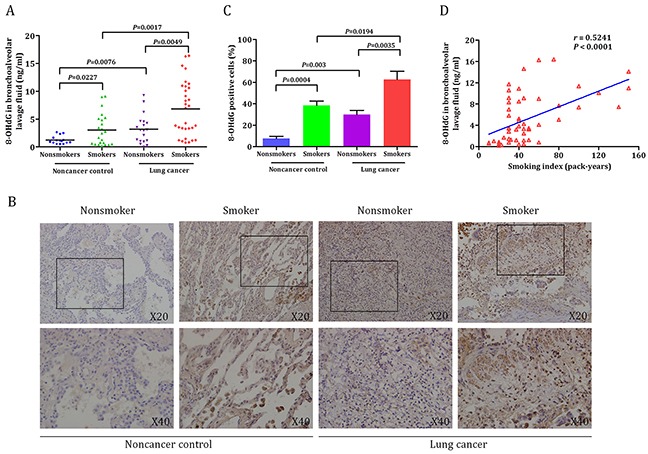
The relationship between smoking and oxidative DNA damage response **A.** The levels of 8-OHdG in BALF was significant higher in smokers than nonsmokers both in lung cancer patients (6.8 ± 0.9 ng/ml *versus* 3.2 ± 0.6 ng/ml, *P* = 0.0049) and noncancer controls (3.0 ± 0.6 ng/ml *versus* 1.2 ± 0.2 ng/ml, *P* = 0.0227). **B.** Representative examples of immunohistochemistry of 8-OHdG expression in smokers and nonsmokers. **C.** Quantification of 8-OHdG expression in the lung of smoking and nonsmoking patients with lung cancer (62.7 ± 7.7 % *versus* 29.8 ± 4.0 %, *P* = 0.0035) and noncancer controls (38.5 ± 4.1% *versus* 7.5 ± 2.2 %, *P* = 0.0004). **D.** Statistically correlation was observed between levels of 8-OHdG and smoking index (r = 0.5241, *P* < 0.0001). Values in the bar graphs are given as mean ± SEM; BALF, bronchoalveolar lavage fluid.

To identify whether oxidative DNA damage was associated with smoking, the levels of 8-OHdG in BALF were analyzed according to smoking status (Figure 3A). Our data indicated that the levels of 8-OHdG were significant higher in smokers than nonsmokers both in lung cancer patients (6.8 ± 0.9 ng/ml *versus* 3.2 ± 0.6 ng/ml, *P* = 0.0049) and noncancer controls (3.0 ± 0.6 ng/ml *versus* 1.2 ± 0.2 ng/ml, *P* = 0.0227). There finding were consistent with IHC staining results (Figure 3B). Statistically significant results were obtained in the lung tissues of smoking and nonsmoking patients with lung cancer and noncancer controls (Figure 3C). To determine how smoking behaved in oxidative DNA damage response, we made scatter plots of BALF 8-OHdG levels and smoking index (Figure 3D). The data showed a statistical significance correlation between the levels of 8-OHdG in BALF and smoking index, with a correlation coefficient (r) of 0.5241. Taken together, these data underscore the importance of smoking in oxidative DNA damage response.

### Diagnostic value of BALF 8-OHdG in lung cancer

**Figure 4:**
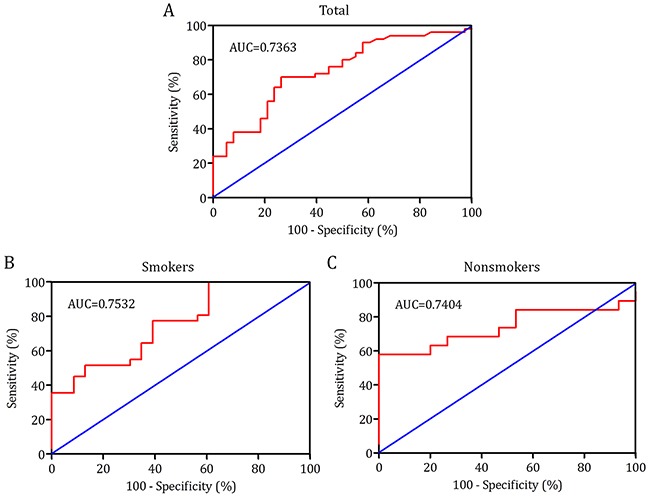
Receiver operating characteristic (ROC) curve was performed to evaluate the threshold value of 8-OHdG in differentiating lung cancer from benign diseases **A.** With a cut-off value of 2.86 pg/ml, 8-OHdG reached a sensitivity of 70.0%, 95% CI (55.4%, 82.1%), a specificity of 73.7%, 95% CI (56.9%, 86.6%). The area under the curve (AUC) was 0.7363, 95% CI (0.6323, 0.8404). **B–C.** According to ROC analysis, the cut-off levels of 8-OHdG in BALF for smokers and nonsmokers was 3.23 ng/ml and 1.885 ng/ml, respectively, and the corresponding sensitivity and specificity were 77.4% (95% CI, 58.9%-90.4%) and 60.9% (95% CI, 38.5%-80.3%), 63.2% (95% CI, 38.4%-83.7%) and 80.0% (95% CI, 51.9%-95.7%), respectively. Additionally, AUC for smokers and nonsmokers was 0.7532 (95% CI, 0.6246-0.8817) and 0.7404 (95% CI, 0.5662-0.9145), respectively.

Fiberoptic bronchoscopy is regularly performed when patients suspected of having lung cancer, during which bronchial washing are traditionally used. In our previous reports, we observed that detection of biomarkers in BALF can serve as an important method for lung cancer diagnosis [4–6]. The present study demonstrated that 8-OHdG was a biomarker for lung cancer. Then, we assessed the usefulness of 8-OHdG in BALF for differential diagnosis of pulmonary malignancy. The diagnostic value of BALF 8-OHdG for lung cancer was evaluated by receiver operating characteristic (ROC) curves analysis (Table 2). The results revealed that levels of 8-OHdG were robust in discriminating patients with lung cancer from noncancer controls with an area under the curves (AUC) value of 0.7363 (95% CI, 0.6323-0.8404) (Figure 4A). Using a cut-off value of 2.86 ng/ml, the sensitivity and specificity predictive values were 70.0% (95% CI, 55.4%-82.1%) and 73.7% (95% CI, 56.9%-86.6%), respectively, to identify a patient with lung cancer. To generate the optimum cutoff score, we further performed ROC curve by smoking status. Comparing lung cancer patients with noncancer group, the best cutoff level of 8-OHdG in BALF of smokers and nonsmokers was 3.23 ng/ml and 1.885 ng/ml, corresponding AUC was 0.7532 (95% CI, 0.6246-0.8817) (Figure 4B) and 0.7404 (95% CI, 0.5662-0.9145) (Figure 4C), respectively. The sensitivity and specificity were 77.4% (95% CI, 58.9%-90.4%) and 60.9% (95% CI, 38.5%-80.3%) for smokers, and 63.2% (95% CI, 38.4%-83.7%) and 80.0% (95% CI, 51.9%-95.7%) for nonsmokers.

**Table 2:** Diagnostic value of BALF 8-OHdG in lung cancer

	AUC	Cut-off value (ng/ml)	Sensitivity (%)	Specificity (%)
Total	0.7363 (0.6323,0.8404)	2.86	70.0% (55.4%,82.1%)	73.7% (56.9%,86.6%)
Smokers	0.7532 (0.6246,0.8817)	3.23	77.4% (58.9%,90.4%)	60.9% (38.5%,80.3%)
Nonsmokers	0.7407 (0.5662,0.9145)	1.885	63.2% (38.4%,83.7%)	80.0% (51.9%,95.7%)

BALF, bronchoalveolar lavage fluid; 8-OHdG, 8-Hydroxydeoxyguanosine; AUC, area under the curves.

## DISCUSSION

The present study showed a higher 8-OHdG expression and secretion in airways of lung cancer patients than that of noncancer controls, which was agreed with previous findings in peripheral blood and urinary [21–23]. Interesting, the levels of 8-OHdG in BALF were associated with the TNM stage, which indicated that oxidative DNA damage was an indicator for the development of lung cancer. Additionally, subgroup analysis by tumor histology, a statistically significant difference in BALF 8-OHdG concentration was found between different cell type of lung cancer patients and patients with benign diseases. Taken together, these findings suggested that 8-OHdG could possibly behave as a biomarker for lung cancer.

It should be noted that a positive correlation was existed between cigarette smoke and oxidative DNA damage response. Cigarette smoke-induced oxidative DNA damage response was observed in bronchial epithelial cells *in vitro* and *in vivo*. In human samples, the expression of 8-OHdG was significant higher in smokers than nonsmokers both in lung cancer patients and noncancer controls. Moreover, a statistical significance correlation was found between the levels of 8-OHdG in BALF and smoking index, with a correlation coefficient (r) of 0.5241. These findings underscore the importance of smoking in oxidative DNA damage.

Our study indicated that 8-OHdG could be of significant clinical value in lung cancer. Then, we assessed the usefulness of 8-OHdG in BALF for differential diagnosis of pulmonary malignancy. ROC analysis revealed that levels of 8-OHdG were robust in discriminating patients with lung cancer from noncancer controls with an AUC value of 0.7363. Using a cut-off value of 2.86 ng/ml, the sensitivity and specificity predictive values were 70.0% and 73.7%, respectively, to identify a patient with lung cancer. In addition, the best cutoff level of 8-OHdG in BALF of smokers and nonsmokers for lung cancer was 3.23 ng/ml and 1.885 ng/ml, corresponding sensitivity and specificity were 77.4% and 60.9%, and 63.2% and 80.0%, respectively.

Several studies were conducted to investigate oxidative DNA damage response in lung cancer in recent years [21–25]. Compared with previous articles, our study provided the new evidence in three ways. Firstly, most previous studies investigated the levels of 8-OHdG in peripheral blood and urinary of lung cancer patients. Our study firstly assessed the levels of 8-OHdG in BALF and our findings can directly reflect the oxidative DNA damage response in airways. Secondly, our study has explored the relationship between smoking and oxidative DNA damage response in airways of lung cancer. Thirdly, to the best of our knowledge, our study is the first study to date that has assessed the utility of 8-OHdG in airways for differential diagnosis of lung cancer. However, some limitations of our study are also worth discussing. First, the underlying mechanisms of how cigarette smoke induced oxidative DNA damage response in airways are still unknown. Second, the sample size in the subgroup analysis was not large enough. Additional studies with larger sample sizes are warranted to validate our findings.

Overall, the findings from our study may have several clinical implications. Firstly, the results from our study may help us to understand oxidative DNA damage response in patients with lung cancer. Secondly, our study underscored the importance of smoking in oxidative DNA damage response, which might lead to smoking avoidance strategies in high risk individuals. Most importantly, we have demonstrated that 8-OHdG was a useful biomarker for lung cancer.

Conclusively, this study showed that cigarette smoke was associated with oxidative DNA damage response in the lung. In addition, 8-OHdG can serve as a useful biomarker for lung cancer. Detection of 8-OHdG in BALF might be helpful for differential diagnosis of lung cancer.

## MATERIALS AND METHODS

### Patients and specimens

88 patients with suspected lung cancer and undergo bronchoscopy in the Affiliated Hospital of Ningbo University were enrolled in this study. Clinical information regarding patient characteristics was based on patient records and registries. Furthermore, 30 lung tissue samples were collected for IHC study. All patients had histological confirmed and were excluded if they had undergone radiotherapy or chemotherapy; or had a previous history of other cancer. Tumors were classified as stage I ∼ IV according to the guidelines of the 7^th^ edition of TNM staging in lung cancer [26]. Approval for this study was obtained from the local ethics committee, and informed consent was obtained from all participating subjects.

### Cell culture

HBE cells were cultured in RPMI 1640 (Sigma-Aldrich, St. Louis, USA) supplemented with 10% FBS at 37°C in a humid atmosphere of 5% CO_2_. The culture medium was changed daily.

### Mice model

C57BL/6 mice (male, aged 8-10 wk old) were purchased obtained from Slac Laboratory Animal Center (Shanghai, China). The mice were exposed to cigarettes in a chamber using a smoking machine (Model TE-10, Teague Enterprises). Mice were exposed 5 d/week for 12 weeks or 24 weeks. The control group was exposed to filtered air under identical conditions. The study protocols were approved by the Ethics Committee for Animal Studies of Zhejiang University, China.

### Bronchoalveolar lavage

The lavage was done prior to brushing or biopsies to avoid contamination with blood. After the local upper airways were anesthetized with 5 mL of 2% lidocaine, the bronchus on the disease side was washed with two 50-ml aliquots sterile physiological saline. The fluid was gently withdrawn into a siliconized container placed in iced water. The lavage fluid was filtered through a nylon filter to remove mucus and centrifuged at 3,000 rpm for 10-min. The cell pellets were separated from the supernatants and stored at −80°C.

### ELISA

The levels of 8-OHdG (ng/ml) were measured using Quantikine sandwich enzyme linked immunosorbent assays (ELISA; Cell Biolabs Inc, San Diego, CA, USA). The assays were conducted according to the manufacturer's guidelines. The kit has an 8-OHdG detection sensitivity range of 100 pg/mL to 20 ng/mL.

### Immunohistochemistry

Paraffin-embedded tissues were cut into 3-5-μm sections for dewaxing and rehydration. The tissue sections were incubated with primary antibody (anti-8-OHdG mouse monoclonal antibody, 1:200, Abcam, Cambridge, UK) overnight at 4°C. After washing with PBS, slides were incubated for 1hr with secondary antibody. Staining slides were read on an Olympus optical microscope over yellow-brown color stains for 12 consecutive fields and scored according to the number of positively stained cells. All immunohistochemical staining results were scored by two observers.

### Immunofluorescence

HBE cells were seeded on coverslips in 24-well plates and treated with or without CSE. The cells were fixed in 4% paraformaldehyde and permeabilized with 0.2% Triton X-100. After blocking with 5% bovine serum albuminin PBS, the cells were incubated with primary antibody overnight at 4°C, and then incubated with secondary antibody diluted in PBS containing 5% bovine serum albumin. DNA was counterstained with 1 mg/ml 4′,6-diamidino-2-phenylindole (DAPI).

### Flow cytometry analysis

After CSE treatment, HBE cells were collected and stained with 8-OHdG, and flow cytometry assays were performed according to the manufacturer's instructions. Data were acquired with a FACS Calibur flow cytometer (BD FACS Calibur, San Jose, CA) and analyzed using FlowJo software.

### Statistical analysis

The analyses were performed using SPSS version 13.0 (SPSS, Chicago, IL, USA) and GraphPad Prism 5.0 (GraphPad Software, San Diego, CA, USA). Data was presented as means ± standard error of the mean (SEM). Comparison between different groups was done using the Student's *t*-test. The relationship between smoking index and BALF 8-OHdG levels was assessed by Pearson correlation. ROC curves were constructed to determine the diagnostic performance of 8-OHdG levels in distinguishing patients with lung cancer from noncancer individuals. Sensitivity against the percentage of false-positives (100-specificity) was plotted each cutoff threshold, and AUC values that reflect the probability of correctly identifying lung cancer patients from noncancer subjects were calculated. All *P*-values were determined from 2-sided tests, and statistical significance was based on a *P*-value < 0.05.
